# Dacryoendoscopy in patients with lacrimal outflow obstruction: a systematic review

**DOI:** 10.1007/s10792-024-03388-z

**Published:** 2025-03-14

**Authors:** Nicole Tsz Yan Wong, Fatema Mohamed Ali Abdulla Aljufairi, Kenneth Ka Hei Lai, Joyce Kar Yee Chin, Clement Chee Yung Tham, Chi Pui Pang, Kelvin Kam Lung Chong

**Affiliations:** 1 Department of Ophthalmology and Visual Sciences, Faculty of Medicine, The Chinese University of Hong Kong, 4/F, Hong Kong Eye Hospital, 147K Argyle Street, Kowloon, Hong Kong Special Administrative Region China; 2 Department of Ophthalmology and Visual Sciences, Prince of Wales Hospital, Sha Tin, Hong Kong Special Administrative Region China; 3 Department of Ophthalmology, Salmaniya Medical Complex, Government Hospitals, Manama, Bahrain; 4 Department of Ophthalmology, Tung Wah Eastern Hospital, Sheung Wan, Hong Kong Special Administrative Region China; 5 Department of Ophthalmology, Hong Kong Eye Hospital, Kowloon, Hong Kong Special Administrative Region China; 6 Eye Centre, The Chinese University of Medical Centre, Sha Tin, Hong Kong Special Administrative Region China

**Keywords:** Dacryoendoscope, Dacryoendoscopy, Endoluminal lacrimal duct recanalization, Endocanalicular lacrimal duct recanalization, Endoscopic lacrimal duct recanalization

## Abstract

**Purpose:**

Dacryoendoscopy (DE) is an emerging, minimally invasive, surgical technique for lacrimal outflow obstruction (LOO). This is a systematic review on the diagnostic and therapeutic use, as well as the safety of DE in the lacrimal literature.

**Methods:**

Up to 22nd November, 2024, 259 studies were retrieved from PubMed, Cochrane Library, and Ovid MEDLINE. After removing duplicates and applying the selection criteria, 18 eligible studies were included. A risk of bias assessment was conducted. The primary outcomes included diagnostic accuracies, therapeutic outcomes, and treatment-related complications. The specifications of DE, lacrimal stents, key procedural steps, and the use of operative adjuvants were also evaluated.

**Results:**

The DE provides additional endoluminal information for LOO by identifying the type (structural or secretory), the location (pre-sac or post-sac), and the pattern (focal or diffuse) of obstruction, while these parameters varied across studies. Notably, pressure-controlled, air-insufflated, high-definition DE (HDDE) provides significantly better image quality than saline-infused system. DE demonstrated variable therapeutic success, both objectively (anatomical and functional patency), and subjectively (symptomatic improvement). The use of adjuvants, such as ballon dacryoplasty, intubation, postoperative topical steroids, antibiotics, and irrigation, appeared to enhance the therapeutic outcomes. Complications were generally mild to minimum, with false passages being the commonest. DE manufactured in Japan and the “Nunchaku-type” of silicone tubes were most commonly used in the literature.

**Conclusions:**

With instrumental advances and endoscopic experience, DE showed potential and early promises to be a minimal invasive alternative for diagnosing and treating LOO of different etiologies.

**Supplementary Information:**

The online version contains supplementary material available at 10.1007/s10792-024-03388-z.

## Introduction

Lacrimal outflow obstruction (LOO) commonly presents as epiphora or mucopurulent discharge, which can be further complicated by dacryocystitis, mucocele and may directly or indirectly affect vision [1]. The current gold standard of treatment for LOO include both external dacryocystorhinostomy (DCR) (EXT-DCR), which involves the removal of maxillary and lacrimal bones to create an intranasal ostium by suturing flaps of lacrimal sac to those of nasal mucosa through a cutaneous incision [2], with reportedly high success rate ranging from 80 to 99% [3–10], or endonasal dacryocystorhinostomy (END-DCR), creating the same intranasal ostium, often without suturing, through an endonasal approach which is skin, orbicularis and thus lacrimal pump-preserving [11–15]. Other methods for treating LOO include “blinded” probing [16], silicone tube intubation (STI) [17, 18], and balloon dacryoplasty [19, 20].

Recently, the dacryoendoscopy (DE)-guided recanalization procedure, also known as endoluminal lacrimal duct (LD) recanalization (ELDR) or endocanalicular LD recanalization, has emerged as a minimally invasive surgical option for LOO. DE allows direct visualization of the lacrimal drainage system (LDS). The diagnostic accuracy and therapeutic outcome of DE-guided examination and/or recanalization in pediatric subjects with congenital nasolacrimal duct obstruction (CNLDO) to adult subjects with primary acquired nasolacrimal duct obstruction (PANDO) or canalicular obstructions (CO), have been increasingly reported.

The aim of this systematic review is to comprehensively study the diagnostic and therapeutic roles of DE in LOO, as well as its safety. We also compare the instruments used including the specifications of DE and lacrimal stents, the techniques for DE-guided recanalization, diagnostic and therapeutic outcome measures, the use of adjuvants, and treatment-related complications reported in the literature. The review seeks to evaluate the benefits and risks of DE-guided procedures, focusing on its accuracy, efficacy, complications with respect to its divergent potential indications.

## Methodology

Our systematic review followed the PRISMA 2020 guidelines [21].

### Search strategy

Up to 22th November, 2024 we conducted our literature search on the following electronic bibliographic databases: PubMed, Cochrane library, and Ovid Medline. We formulated sensitive search strategies using keywords stated in Table S1. No language restrictions or limitations on the publication years were applied. A total of 259 results were retrieved (130 from PubMed, 4 from Cochrane Library, 125 from Ovid Medline). 14 duplicates were identified, and 245 results were left for screening (Fig. S1).

### Eligibility criteria

The search primarily focused on identifying literature reporting the use of DE in patients with LOO. Among the 245 studies, we included those based on the following inclusion criteria: 1) studies that evaluated the use of DE in patients with different types of LOO including CO, and 2) studies that were observational case series, case–control studies, cohort studies, interventional case series, or clinical trials. Animal studies, case reports, reviews, registered trials, abstracts, and studies published in foreign languages were excluded.

In the first phase of the review, the titles and abstracts were screened by three independent reviewers (N.W., F.A., and K.L.) after applying the search strategy and eligibility criteria. Disagreements were resolved through discussions with two senior reviewers (J.C. and K.C.). 189 irrelevant results were removed. Full-text screening was then performed on the remaining 56 eligible articles by three independent reviewers (N.W., F.A., and K.L.). Disagreements were again resolved through discussions with the senior reviewers (J.C. and K.C.). After ensuring eligibility, a total of 18 studies were included in the review for analysis (Fig. S1).

### Literature review

We used a pre-designed form to collect all necessary information, including the study title, year of publication, authors’ names, country of study, ethnicity, study design, sample size, patient condition or definition, age, gender, follow-up (FU) duration, types of instruments used (mainly DE and stents), DE procedures or techniques, outcome measures (mainly diagnostic, therapeutic, or both), adjuvants used, and complications.

Data were extracted by three independent reviewers (N.W., F.A., and K.L.), and discrepancies were resolved through discussions with two senior reviewers (J.C. and K.C.).

### Assessment of risk of bias, study quality, and level of evidence

We used the Cochrane Risk of Bias Tool (Rob 2) as outlined in Chapter 8 of the Cochrane Handbook for Systematic Review of Interventions to assess the risks of bias of randomized-controlled trial (RCT) [22]. We evaluated the five domains: randomization process, intervention deviations, missing outcome data, outcome measurement, and selection of results. The overall risk of bias was categorized as “low risk”, “some concerns”, or “high risk” based on the suggested algorithm.

In preparing the Preferred Practice Pattern (PPP) guideline [23], we adopted the rating system used by the American Academy of Ophthalmology, which incorporates elements of the Scottish Intercollegiate Guideline Network (SIGN) and Grading of Recommendations Assessment, Development, and Evaluation (GRADE) frameworks. The level of evidence was categorized into three levels: Level I included randomized controlled trials (RCTs) with a low risk of bias, systematic reviews of RCTs, or meta-analyses; Level II included well-designed case–control or cohort studies; and Level III included non-analytic studies such as case reports or case series. Recommendations for care were rated as follows: Level A indicated the most important applications to care; Level B indicated moderately important applications to care; and Level C indicated relevant but less critical applications to care.

Three independent reviewers (N.W., F.A., and K.L.) performed the quality assessment. Disagreements were resolved through discussions with the two senior reviewers (J.C. and K.C.).

## Results

From our literature search, we identified a total of 259 titles and abstracts, and retrieved 56 full texts for review. We finally included 18 studies in our systematic review [24–41]. Table S2 summarizes the results of our review.

### Characteristics of studies

Table S3 summarizes the characteristics of the 18 included studies [24–41]. Of these, 15 (83.3%) studies were conducted in patients with Asian ancestry [24, 26–32, 34–37, 39–41], including four (22.2%) in Korea [24, 32, 34, 35], eight (53.3%) in Japan [28–30, 37, 39–41], two (11.1%) in Philippines [31, 36], and one (5.6%) in China [26]. Two (11.1%) studies were conducted in patients with Indian ancestry [33, 38]; and one (5.6%) in patients with Caucasian ancestry in Germany [25].

In terms of study design, 13 (72.2%) studies were retrospective case series [24–35, 41], four (22.2%) were prospective case series [37–40], and one (5.6%) was RCT [36]. Regarding the sample sizes, 14 (77.8%) studies reported the total number of eyes studied [24–26, 29–36, 39–41], which ranged from 13 eyes [29, 33] to 203 eyes [24]. The remaining four (22.2%) studies only reported the total number of patients studied [27, 28, 37, 38], which ranged from 26 patients [38] to 128 patients [28]. The studies also varied in terms of the targeted age groups: eight (53.3%) studies focused on pediatric populations [25, 26, 29, 30, 33, 38, 39, 41], with ages ranging from 1–12 months [25] to 14–74 months [29]; while ten (55.6%) studies focused on adult populations [24, 27, 28, 31, 32, 34–37, 40], with ages ranging from 17–77 years [31] to 31–102 years [40]. Gender distribution was reported in all studies except for two [38, 39], with female representation ranging from 30.8% [33] to 100% [32] and male representation ranging from 0% [32] to 69.2% [33].

The patient groups varied across the studies: nine (50%) studies recruited patients with PANDO [24, 27, 28, 31, 32, 34–36, 40], three (33.3%) of which also included patients with CO [27, 28, 35], and two (22.2%) specifically focused on patients with failed previous probing [32, 34]. Eight (44.4%) studies recruited patients with CNLDO [25, 26, 29, 30, 33, 38, 39, 41], three of which focused on patients with failed previous probing [26, 30, 33]. One study recruited patients with lacrimal outflow obstruction-not otherwise specified (LOO-NOS) [37].

FU duration was available in all studies except three [27, 38, 40], spanning from as short as 2 weeks [41] to as long as 24.8 ± 15.5 months [25]. In terms of study outcomes, two (11.1%) studies were diagnostic [27, 40]; six (33.3%) studies were therapeutic [28, 29, 31, 33, 35, 36]; ten (55.6%) studies were both diagnostic and therapeutic in nature [24–26, 30, 32, 34, 37–39, 41].

### Assessment of study quality and grading of clinical recommendation

Table S4 summarizes the assessment of study quality including both the level of evidence and grading of clinical care recommendation of the included studies. Javate et al. [36] had the highest level of evidence among the other studies, while all other studies were given a Level III strength of evidence and Level B importance of application. For the RCT [36], its risk of bias was assessed using the Rob2 Tool. We rated “some concerns” for both the randomization process and outcome measurement due to the lack of details of the stated “completely randomized design” and the awareness of intervention by the assessor respectively, and “low risk” for intervention deviation, missing outcome data, and result selection. The overall risk of bias was “some concerns”.

### Instruments used

**(1) Types of dacryoendoscope**

According to the 18 studies, 13 (72.2%) of the DE used were manufactured in Japan [24, 27–32, 34, 35, 37, 39–41] while five (29.4%) were made in Germany [25, 26, 33, 36, 38]. Among the 13 DE made in Japan, 12 (92.3%) of them adopted the DE by Fibertech Co., Tokyo, Japan [24, 27–30, 32, 34, 35, 37, 39–41], while only one (7.7%) study [31] adopted that manufactured by Machida Endoscope Co., Ltd., Japan. Regarding the three-charge-couple device (3CCD) imaging system used, Fibertech Co. clearly stated the model used, in which seven studies [24, 27, 29, 30, 32, 34, 35] used DE coupled with FT-203F, two studies [37, 40] used FT-2000E, one study [28] used FT-201, one study [39] used MD10, and one study [41] used MT-3000. In terms of physical characteristics, the DE manufactured by Fibertech Co. had a shorter probe length (50 mm [24, 29, 30, 32, 34, 35, 41] versus 60 mm [31]), a larger probe outer diameter (0.9 mm [24, 27, 30, 32, 34, 35, 39, 40] versus 0.7 mm [31]), a wider field of view (70° [24, 30, 32, 34, 35, 40] versus 65° [31]), more image elements (6000 [24, 30, 32, 34, 35, 40] or 10,000 [27, 39] versus 5000 [31]), and a greater observation depth (10 mm [24, 29, 30, 32, 34, 35, 40] vs 5 mm [31]) than Machida Endoscope Co., Ltd.. In terms of physical characteristics, the DE manufactured by Fibertech Co. had a shorter probe length (50 mm [24, 29, 30, 32, 34, 35, 41] versus 60 mm [31]), a larger probe outer diameter (0.9 mm [24, 27, 30, 32, 34, 35, 39, 40] versus 0.7 mm [31]), a wider field of view (70° [24, 30, 32, 34, 35, 40] versus 65° [31]), more image elements (6000 [24, 30, 32, 34, 35, 40] or 10,000 [27, 39] versus 5000 [31]), and a greater observation depth (10 mm [24, 29, 30, 32, 34, 35, 40] vs 5 mm [31]) than Machida Endoscope Co., Ltd.. Among the five DE devices made in Germany, three (60%) were manufactured by Karl Storz GmbH and Co., Tuttlingen, Germany [33, 36, 38]], and two (40%) were manufactured by Polydiagnost GmbH, Germany [25, 26]. The German DE devices used different imaging systems; the high definition IMAGE1 STM Karl Storz imaging system [33, 38] and the Karl Storz AIDA compact II System [36]. However, these German devices had similar probe outer diameter (0.75–0.9 mm [25, 33, 38]), field of view (70° [25]), and image elements (6000 [25]) as the Japanese devices. Most of the DE devices manufactured by German companies had 0° direction [25, 33, 36, 38], while most DE devices manufactured by Japanese companies had 27° probe bending angle [24, 29, 30, 32, 34, 35, 40, 41].

**(2) Types of stents**

According to the 18 studies, nine (50%) studies [24, 25, 28, 31, 32, 34, 35, 37, 39] specified the manufacturers of the silicone tubes (STs) or stents used for intubation of LDS. These included four studies using stents made in South Korea by Yuwon Meditech Co., Ltd. [24, 32, 34, 35], three studies using stents made in Japan by Kaneka Co., Ltd. [37, 39] and NIDEK Co., Ltd. [28], and two studies using stents made in France by FCI, Paris [25, 31]. Among them, five (55.6%) studies also specified the types of stents used. These included Bika [35], Lacrifast [39], Crawford [31], Ritleng + [31], MonokaÒ [25], and Nunchaku [31, 37]. All of these stents were bicanalicular stents, while the Nunchaku STs specifically noted to be easier to insert, more stable and flexible, and capable of preventing the need for stent retrieval, making them less invasive compared to the other stent types [31, 37].

### Procedures of DE-guided recanalization

The DE-guided recanalization operation was generally similar across the 18 studies, though there were some differences in technique.

**(1) General procedure**

 The procedure typically involved nasal packing for vasoconstriction of the nasal mucosa, followed by the administration of either general, local, or topical anesthesia, depending on the patient’s needs. A nasoendoscope (NE) was inserted at the inferior meatus (IM) to facilitate access and visualization of the nasolacrimal duct (NLD) opening. A punctum dilator was used to dilate the lacrimal puncta; upper, lower, or both. Then a DE, which was connected to an imaging system, was inserted through the upper, lower, or both lacrimal puncta. Saline was irrigated through the water channel of the DE to create positive pressure, remove blockades, and improve visibility of the lumen. If an obstruction was encountered, recanalization could be done by direct endoscopic probing, where the DE tip was advanced to perforate the obstruction, or by sheath-guided endoscopic probing, where a customized DE sheath, pre-loaded onto the DE, was used to release the obstruction. A stent was then inserted to prevent re-obstruction, and luminal patency was confirmed using the DE and NE, indicated by the smooth passage of saline [24, 25].

**(2) Differences**

 Minor variations in technique existed across the studies. Two studies used a free elevator with the NE to mobilize the nasal turbinate for better access and visualization of the NLD opening [31, 36]. Instead of direct or sheath-guided endoscopic probing, two studies used a lacrimal trephine, which acted like the DE sheath to assist with probing and perforating the obstruction [31, 36]. A lacrimal trephine offered additional benefits when dealing with dense and fibrotic obstructions due to its rigidity compared to plastic sheaths [31]. One study by Li et al. [26] employed a sickle knife followed by mucosa cutters or superfine mucosal scissors for the incision of Hasner’s valve in refractory membranous CNLDO. Sasaki et al. [27] compared air-insufflated high-definition DE (HDDE) with saline-irrigated HDDE and saline-irrigated conventional DE (CDE), using a pressure-controlled air-insufflation system (0–20 kPa) in place of saline irrigation. Lastly, Javate [31] incorporated balloon dacryoplasty prior to STI, where a balloon catheter was inflated and deflated at two positions within the NLD to dilate the lumen.

### Outcome measures

**(1) Diagnostic ability of DE**

 A total of 12 out of 18 (66.7%) studies investigated the diagnostic use of DE, including the types or causes, locations and patterns of LOO [24–27, 30, 32, 34, 37–41].

**(a) Types or causes of LOO**

 Six out of 18 (33.3%) studies reported that DE could visually identify the types or causes of LOO [24, 25, 28,26]. The types of obstructions detected included stenosis [24, 25, 32, 34, 39] (2.7% [24] to 42.1% [32]), fibrosis or fibrotic membranes [24, 25, 32, 34, 39] (13% [39] to 61% [39]), mucus [24, 32, 34] (15.8% [32] to 39.9% [24]), dacryoliths [24, 25, 32, 34, 39] (3.3% [34] to 11.1% [25]), granulations [24, 32, 34] (8.9% [24] to 13.1% [34]), clefts [39] (13%), or other physical blockages like cystic bulging of Hasner’s membrane (22.2% [25]). Of the studies that classified LOO, three [24, 32, 34] used structural (stenosis, fibrosis) (41.9% [24] to 65.6% [34]) or secretory (mucus, stones, granulations) (34.4% [34] to 58.1% [24]) types, while another classified LOO into simple (membrane, cleft) (73%) or complicated (stenosis, fibrosis) (27%) types [39]. Additionally, DE was useful in identifying the causes of probing failure in patients with CNLDO, where 69.2% of patients showed injuries such as inflammation, scars, adhesions and false passages [26].

**(b) Locations of LOO**

 Eight out of the 18 (44.4%) studies demonstrated that DE could identify the locations of LOO [26, 30, 32, 34, 37, 38, 40, 41]. The locations identified by DE included

1. The canaliculi, appearing whitish to pinkish with folds at various sites.
2. The canalicular-sac junction, characterized by a widening of the lumen.
3. The lacrimal sac, lined with pinkish to reddish lining and covered with thick discharges.
4. The sac-duct junction, where the proximal NLD opening was visible from the medial side of the inferior part of the sac.
5. The NLD, divided into proximal, middle, and distal segments. The distal end was identified by a dilated, bulging membrane with a light pink hue and saline collection [38].
  (3) The locations of LOO were classified as either pre-sac (canaliculus, sac) or post-sac (NLD, IM) [32, 34], or as lower (membranous) or higher (sac-duct junction) [40]. Studies found that more refractory LOO [34] and false passages [32] were primarily located in the pre-sac area in 57.4% [34] and 63.2% [32] of cases, while 42.6% [34] and 36.8% [32] were located at the post-sac area.

**(c) Patterns of LOO**

 One out of 18 (5.6%) studies reported that DE could identify the patterns of LOO [34]. In this study, among 57 patients who failed conventional STI and underwent repeated STI by DE, 77.2% had focal LOO and 22.8% had diffuse LOO.

**(d) Types of DE with better image quality** Most studies used saline-irrigated DE [24, 25, 28–41]. Sasaki et al. [27] compared image quality between air-insufflated HDDE, saline-irrigated HDDE, and saline-irrigated CDE. Air-insufflated HDDE provided the clearest images, especially for identifying obstructed sites. Air-insufflated HDDE were comparable in quality to microscopic images used in DCR in terms of tumor detection. Among the saline-irrigated DE, CDE provided the vaguest images.

In in vivo experiments, air-insufflated DE at lower air pressures (5 kPa) allowed visualization of vascular pulsation and canalicular or sac movement. Higher air pressures (15–20 kPa) extended the duct, providing better avascular images. Low air pressures (below 4 kPa) limited space for DE insertion, leading to inadequate observation [27].

**(2) Therapeutic success of DE**

A total of 16 out of 18 (88.9%) studies investigated the therapeutic aspect of DE [24–26, 28–39, 41]. These studies varied in how they defined therapeutic success, but the outcomes could generally be classified into objective and subjective improvements.

Objective improvement was assessed through both anatomical and functional patency. Anatomical patency ((1) in Table S2) was typically evaluated using the lacrimal irrigation test (LIT) [24, 31, 32, 34–37], or the absence of mucus regurgitation after lacrimal sac compression [25, 36]. Functional patency ((2) in Table S2) was evaluated using a variety of tests, including fluorescein dye disappearance test (FDDT) [25, 26, 30, 35, 37, 41], functional endoscopic dye test (FEDT) [31], fluorescein dye retention test (FDRT) [39], Jones test [36], and tear meniscus height (TMH) [24].

Subjective improvement ((3) in Table S2) was primarily evaluated through patient-reported outcomes, which included changes in symptoms such as the frequency of epiphora as measured by Munk’s scores, or through interviews and questionnaires.

The studies varied in how they defined therapeutic success. Eleven (68.8%) [24–26, 30, 32–35, 37, 39, 41] studies clearly defined therapeutic success using both objective and subjective criteria. Two (12.5%) [31, 36] studies reported objective and subjective success rates separately. One (6.3%) [29] study focused solely on subjective success, while two (12.5%) [28, 38] studies did not provide a clear definition of therapeutic success at all.

**(a) Overall success rates**

Among the 16 studies, 14 (87.5%) [24–26, 28–30, 32–35, 37–39, 41] reported overall high success rates for DE, ranging from 73.7% [32] to 100% [26, 33, 39, 41]. However, two (12.5%) [31, 36] studies calculated objective and subjective success separately. Of the 14 studies reporting high success rates, eleven (78.6%) [24–26, 30, 32–35, 37, 39, 41] concluded that therapeutic success involved both objective (anatomical and/or functional patency) and subjective improvement.

**(i) Overall success in pediatric populations**

A total of eight studies (44.4%) [25, 26, 29, 30, 33, 38, 39, 41] focused on the pediatric population and investigated the therapeutic outcome of DE-guided recanalization. Except for one study by Sasaki et al. [29], which only assessed subjective therapeutic success, and another by Gupta et al*.* [38], which did not clearly define the success rate, all other six (75%) studies reported therapeutic success in both objective (anatomical and/or functional patency) and subjective improvement. The reported success rates ranged from 77.8% [25] to 100% [26, 33, 39, 41].

Among these six studies, three (50%) specifically investigated the role of DE in pediatric patients with refractory CNLDO, with therapeutic success rates ranging from 97.1% [30] to 100% [26, 33]. In Li et al.’s study [26], the surgical success, defined as normal FDDT, normal TMH, and the absence of epiphora, reached 100%. Similarly, Gupta et al. [33] reported a 100% success rate for the absence of epiphora, indicating both anatomical and functional patency. Fujimoto et al. [30] found a success rate of 98.1% at two weeks post-operative and 97.1% one year after surgery, defined as normal FDDT findings and the absence of epiphora and mucous discharge.

The remaining three (50%) studies focused on pediatric patients with CNLDO and reported therapeutic success rates ranging from 77.8% [25] to 100% [39, 41]. Matsumura et al. [39] reported a 100% success rate six months post-surgery, defined by normal FDRT findings and absence of epiphora and mucous discharge. Similarly, Ueta et al*.* [41] reported a 100% success rate two weeks post-surgery, defined by normal FDDT findings and the absence of epiphora and mucous discharge. In contrast, Heichel et al. [25] reported a success rate of 77.8%, defined by the absence of mucosal regurgitation after lacrimal sac compression, the absence of high TMH, and a maximal FDDT time of three minutes. Notably, 94.4% of patients in this study demonstrated success in terms of the absence of epiphora and mucous discharge, high TMH and FDDT outcomes. Although Gupta et al*.* [38] did not clearly define therapeutic success, their study reported an overall recanalization success rate of 88.4%.

**(ii) Overall success in adult populations**

A total of eight (44.4%) studies focused on adult population and investigated the therapeutic outcome of DE-guided recanalization [24, 28, 31, 32, 34–37]. Six (75%) [24, 28, 32, 34, 35, 40] of these studies reported therapeutic success in both objective (*i.e.* anatomical and/or functional patency) and subjective improvement, with success rates ranging from 73.7% [32] to 96.9% [28]. However, two studies, by Javate [31] and Javate et al. [36], studied objective and subjective outcomes separately,

Several studies defined therapeutic success similarly. Kim et al. [34], who compared DE-STI and DE-DCR in patients with failed conventional STI, and Lee et al. [24], who investigated DE in patients with PANDO, both defined success as passing the LIT, TMH < 0.3 mm, and a post-operative Munk’s score 0 or 1. The overall success rates in these studies were 83.9% [34] and 86.2% [24], with Kim et al. [34] also noting that DE-STI had a success rate of 93.4%, while DE-DCR achieved a 100% success rate.

Kim et al. [32], who focused on the role of DE in managing false passages in patients with PANDO, found an overall success rate of 73.7%, based on similar criteria: passing the LIT, TMH < 0.3 mm, and a decreased Munk’s score.

Kabata et al. [37], who investigated quality of life in patients with complete and unilateral LOO-NOS, reported a the surgical success rate of 89%, defined by good fluid passage during lacrimal irrigation, dye disappearance in DDT, and symptomatic improvement of epiphora three months post-operatively.

Kim et al. [35], who studied the outcome of DE-guided recanalization in patients with PANDO and CO, defined success as either a complete or partial response. Complete response was defined as (1) good fluid passage during lacrimal irrigation, (2) normal FDDT findings, and (3) absence of epiphora (TMH ≤ 0.3 mm and Munk’s score ≤ 1), while partial response involved either (1) or (3) along with (2). The overall success rate was 89.2%, with 75.7% achieving complete response and 13.5% achieving partial response.

Kamao et al. [28], who assessed both objective and subjective outcomes in patients with pre-sac and post-sac PANDO or other LOO, reported a surgical success rate of 96.9%, although the criteria for success were not clearly defined.

**(iii) Comparisons between different types of LOO**

Both Kim et al. [34] and Lee et al. [24] compared the therapeutic success rates of DE-guided recanalization in patients with refractory PANDO [34] or PANDO [24] in terms of different types (*i.e.* structural vs secretory). They found that those with structural type had a higher success rate than those with secretory type (84.4% versus 66.7%; *P* = 0.015 [34]; 95.3% versus 79.7%%; *P* = 0.001 [24]). Lee et al. [24] also found a 100% success rate in subjects with membranous obstruction (structural type) at IM, regardless of the results of LIT.

However, Kim et al. [32] found that, in patients with PANDO and false passages, the success rates in those with secretory type were higher than those with structural type (85.7% versus 66.7%).

**(iv) Comparisons between different locations of LOO**

Kim et al. [34] compared the therapeutic success rates of DE-guided recanalization in patients with refractory PANDO at different locations (pre-sac vs. post-sac). They found that patients with post-sac or lacrimal sac obstructions had a higher success rate (95.5%) compared to those with at pre-sac obstructions (61.9%), with a significant difference (*P* = 0.034).

Lee et al. [24] also reported a 100% success rate in patients with membranous PANDO at the IM (post-sac), regardless of the results of LIT.

Kim et al. [32] found that, in patients with PANDO and false passages, the success rates were higher in those with pre-sac obstructions at canaliculus (83.3%) compared to those with post-sac obstructions at NLD (71.4%) or lacrimal sac (66.7%).

Kamao et al. [28] assessed the post-operative outcomes in patients with pre-sac and post-sac PANDO or other LOO and reported an 88.7% patency rate, with no significant differences between the two groups. Both groups experienced a significant decrease in TMH, except for those with obstructions at the lacrimal punctum, common lacrimal canaliculus, or NLD.

**(v) Comparisons between different patterns of LOO**

Only Kim et al. [34] and Lee et al. [24] compared the therapeutic success rates of DE-guided recanalization in patients with refractory PANDO [34] or PANDO [24] in terms of different patterns (focal vs diffuse). They found that patients with a focal pattern had higher success rates than those with a diffuse pattern (91.0% versus 61.5%; *P* = 0.022 [34]; 93.1% vs. 74.0%; *P* = 0.003 [24]).

**(vi) Comparisons between different severities of LOO**

Kim et al. [35] investigated the outcome of DE-guided recanalization in patients with PANDO and CO. They found that the success rates in patients with “partial passage” identified in pre-operative LIT were higher than those with “no passage” (95.9% versus 76.0%; *P* = 0.01).

Kim et al. [32] found that the success rates were higher in patients with an absence of past lacrimal treatment history compared to those with a history of past lacrimal treatments (100% versus 70.6%).

Kamao et al. [28] assessed the post-operative outcomes in patients with pre-sac and post-sac PANDO or other LOO and reported an overall 88.7% patency rate. In the pre-sac group, those with complete obstructions and an intermediate duration (1 to 3 years) of epiphora had higher patency rates than those with incomplete obstructions and a short-term (within 1 year) or long-term (more than 3 years) epiphora duration (95.6% versus 90.0%; 100.0% versus 92.9% versus 91.7%). In the post-sac group, patients with incomplete obstructions and a short-term epiphora had higher patency rates than those with complete obstructions and an intermediate or long-term duration of epiphora (100.0% versus 83.1%; 90.9% versus 82.4% versus 77.3%). For pre-sac locations, the overall therapeutic success of more severe LOO remained high, generally more than > 70%.

**(b) Objective therapeutic outcomes**

Apart from the above 14 studies [24–26, 28–30, 32–35, 37–39, 41] which concluded a high (73.7% [32] to 100% [26, 33, 39, 41]) overall therapeutic success of DE-guided recanalization in terms of both objective (anatomical and/or functional patency) and subjective improvement, Javate [31] and Javate et al. [36] further dug into the objective success individually, and found that DE-guided recanalization could achieve great objective improvement in terms of both anatomical and functional patency.

**(i) Anatomical patency**

Javate [31] who incorporated balloon dacryoplasty to STI in patients with complete PANDO found that, the success rate of anatomical patency on canalicular irrigation with saline was 95.6%.

Javate et al. [36] who compared the efficacy of standard external-DCR (SE-DCR) and ELDR in patients with complete or partial PANDO also found that, the combined anatomical patency, defined by patency in lacrimal irrigation test and an absence of mucous regurgitation after lacrimal sac compression, of ELDR and SE-DCR were 93.0% and 93.7% respectively (*P* = 0.85).

**(ii) Functional patency**

Javate [31] who incorporated balloon dacryoplasty to STI in patients with complete PANDO found that, the success rate of functional patency by FEDT was 95.6%.

Javate et al. [36] who compared the efficacy of SE-DCR and ELDR in patients with complete or partial PANDO also found that, the combined functional patency, defined by a positive primary Jones test, of ELDR and SE-DCR were 84.9% and 90.0% respectively (*P* = 0.32).

**(c) Subjective therapeutic outcomes**

Among the 16 studies [24–26, 28–39, 41] that looked into the therapeutic aspect of DE, six (37.5%) [25, 28, 29, 31, 36, 37] of them additionally studied the subjective therapeutic outcomes individually by means of Munk’s score, interviews, and questionaries, which all showed a great subjective improvement achieved by DE-guided recanalization in terms of both symptoms and quality of life.

**(i) Munk’s score or epiphora improvement**

Javate [31] who incorporated balloon dacryoplasty to STI in patients with complete PANDO found that, there was a significant improvement of Munk’s score between pre- and post-DE operation, with pre-operative score being 3 (*P* < 0.001), post-operative score being 0–1 (*P* < 0.001).

Javate et al. [36] who compared the efficacy of SE-DCR and ELDR in patients with complete or partial PANDO monitored any subjective symptomatic improvement of epiphora of patients but did not mention the exact results.

**(ii) Interviews on symptomatic improvement**

Regarding the studies by Heichel et al. [25] and Sasaki et al. [29] who investigated the role of DE in pediatric patients with CNLDO, post-operative interviews were held with subjects’ parents regarding symptoms (epiphora and discharges [25, 29], pain and swelling [29]), 66.7% [25] and 53.8% [29] had excellent results (i.e. absence of symptoms [25, 29]) respectively; while 25% [25] and 38.5% [29] had good results (i.e. clear symptomatic improvement with slight side difference [25] or absence of epiphora with occasional discharge [29]) respectively.

Sasaki et al. [29] also reported the subjective success rate, defined by the absence of epiphora and discharge, being 92.3% (12 out of 13 patients), in which the obstructed site was confirmed by DE in all patients except one.

**(iii) Questionnaires**

Kamao et al. [28] who assessed the post-operative outcomes in patients with pre-sac and post-sac LOO evaluated the subjective outcomes based on the Glasgow Benefit Inventory (GBI) questionnaire and the six-symptom ocular specific questionnaire. The GBI questionnaire consisted of total, general subscale, social support, and physical health scores, in which there were no significant differences between pre-sac and post-sac groups. The six-symptom ocular questionnaire consisted of tearing, discharge, swelling, pain, irritation, and blurred vision, where significant improvement was found in all symptoms (*P* < 0.0001; *P* < 0.0001; *P* = 0.0024; *P* = 0.0002; *P* = 0.0456; *P* = 0.0003). While all sym*P*toms im*P*roved in *P*ost-sac grou*P* (*P* < 0.0001; *P* < 0.0001; *P* = 0.0005; *P* = 0.0011; *P* = 0.0285; *P* = 0.0002), only tearing improved in pre-sac group (P < 0.0001).

Kabata et al. [37] investigated the quality of life in subjects with complete and unilateral LOO-NOS. Besides from evaluating the epiphora improvement as part of the success parameters, they also adopted the National Eye Institute 25-item Visual Function Questionnaire (NEI VFQ-25) as subjective outcome for quality of life at pre-operative and post-operative 3 months. The questionnaire consisted of 12 vision-targeted scales, where significant improvement was reported in the composite score (*P* = 0.0001) obtained by averaging the scores of 11 subscales excluding the general health subscale, ocular pain score (*P* < 0.0001), and mental health score (*P* = 0.0003).

**(3) Use of adjuvants**

A total of nine out of 18 (50%) studies irrigated steroids and antibiotics to LDS at the end of DE procedure and during post-operative period to control inflammation and prevent possible infection [25, 26, 28, 31, 34–37, 41], which in turn contributed to therapeutic success.

**(a) Topical ophthalmic medications only**

Two studies [31, 41] used only topical medications. Javate [31] applied steroid-containing antibiotic eye drops (TobraDex) to the operated eye every three hours on the day of surgery (post-operative day zero). From post-operative day seven onwards, the drops were used weekly for two months, then monthly for four months, and subsequently every three to four months. In contrast, Ueta et al. [41] used antibiotic eye drops for three days post-operatively.

**(b) Systemic medications**

Two studies [34, 35] also used systemic medications. Kim et al. [35] used systemic antibiotics (third generation cephalosporins) and topical antibiotics including eye drops and ointments post-operatively; while Kim et al. [34] used topical antibiotics (levofloxacin) and steroids (fluorometholone) eye drops four times a day for a month, followed by oral antibiotics for a week.

**(c) Nasal medications**

Two other studies [25, 26] used nasal medications besides ophthalmic medications. Li et al. [26] used topical antibiotics (levofloxacin) eye drops for three days and steroids (budesonide) nasal sprays for a week; while Heichel et al. [25] used topical antibiotics and steroids for five times a day and astringent nose drops twice a day in post-operative week one, then only topical antibiotics and steroids three times in post-operative week two.

**(d) Lacrimal duct irrigation**

Meanwhile, three studies [28, 36, 37] irrigated the LD with saline in addition to using medications. Kamao et al. [28] used topical antibiotics (gatifloxacin) and steroids (fluorometholone) four times a day, as well as saline for regular LD irrigation until stent removal; similarly, Kabata et al. [37] used topical antibiotics and steroids eye drops, as well as saline for regular LD irrigation until stent removal 2 months later; Javate et al*.* [36] used topical steroid-containing antibiotics eye drops every three hours and antibiotics ointment during bedtime on post-operative day zero, followed by saline irrigation weekly for a month, where Javate lacrimal dilating cannula was inserted from the upper punctum down to the NLD in case of saline regurgitation.

**(4) Complications**

A total of 14 out of 18 (77.8%) studies concluded that DE had no or few complications only [25–32, 35, 36, 38–41], rendering it a safe procedure.

**(a) No complications**

Eight out of 18 (44.4%) studies reported no complications associated with the use of DE [25], including both early complications [26], such as bleeding [27] or hematoma [31], infection [41], edema [31], inflammation [27], subcutaneous emphysema [27], pain and perforation [40], false passages and damages to surrounding structures [41], as well as late complications [26], such as intubation dislocation and recurrence of stenosis [25].

**(b) Hematoma, edema, inflammation**

Three out of 18 (16.7%) studies reported minor complications such as hematoma, edema, and inflammation in surrounding soft tissues [35, 36, 38]. Javate et al. [36] reported minor complications such as hematoma and edema. Gupta et al. [38] reported one case of edema around the lower eyelid, which resolved within two hours. Kim et al. [35] reported that post-operative inflammation was associated with a lower success rate of DE-guided recanalization and STI, especially in subjects diagnosed with “no passage” rather than “partial passage” by pre-operative lacrimal irrigation test (96.6% versus 60.0%; *p* < 0.001).

**(c) False passages**

Four out of 18 (22.2%) studies found that false passages formation could be one of the few complications of DE [26, 28, 30, 32]; but two of them found that false passages could be managed effectively [24, 32]. Kamao et al. [28] reported that severe fibrosis at the obstructed site may form a false passage, contributing to a 3.1% surgical failure rate. Fujimoto et al. [30] found that 1.9% of patients had false passages in the middle area of NLD. Li et al. [26] found that, despite 69.2% of patients having injuries to the LD, including false passages, the success rate of probing and STI reached 100%. Kim et al. [32] found that 2.3% of patients, especially those with structural rather than secretory type, had false passages, but the overall success rate of STI reached 73.7%.

## Discussions

This systematic review summarizes the current evidence on the use of DE for LOO, covering aspects such as the instruments used, DE-guided recanalization procedures, diagnostic and therapeutic outcomes, adjuvants used, and complications.

The 18 included studies revealed a predominance of research conducted on patients with Asian ancestry (83.3%) [24, 26–32, 34–37, 39–41] , particularly from Japan (53.3%) [28–30, 37, 39–41] and Korea (22.2%) [24, 32, 34, 35]. Most studies (72.2%) utilized retrospective case series designs [24, 25], with fewer prospective case series (22.2%) [37–40] and only one RCT (5.6%) [36]. The sample sizes and population demographics varied widely, reflecting diverse clinical scenarios. Patient groups ranged from pediatric populations (53.5%) [25, 26, 29, 30, 33, 38, 39, 41] to adults (55.6%) [24, 27, 28, 31, 32, 34–37, 40], with conditions such as PANDO (50%) [24, 27, 28, 31, 32, 34–36, 40], CNLDO (44.4%) [25, 26, 29, 30, 33, 38, 39, 41], and previous failed probing (27.8%) [26, 30, 32–34] being studied. While all studies employed DE-guided recanalization, nearly half also incorporated sheath-guided techniques [24, 26–28, 32, 34, 35, 39], highlighting variations in procedural approaches.

The use of DE and stents varied across the 18 studies, reflecting regional and technological differences. 66.7% of the studies employed the DE manufactured by Fibertech Co., Tokyo, Japan [24, 27–30, 32, 34, 35, 37, 39–41]. This device provided superior image quality due to its larger probe outer diameter and smaller image elements, and it caused less damage to the supraorbital margin and medial palpebral ligament during insertion, owing to the probe’s 27° bending angle [34]. Silicone stents were employed for intubation, with manufacturers specified in 50% of the studies [24, 25, 28, 31, 32, 34, 35, 37, 39]. Nunchaku STs were noted to be easier to insert, more stable and flexible. These stents help prevent the need of stent retrieval, making them less invasive than other stent types [31, 37]. DE-guided recanalization provides both diagnostic and therapeutic benefits for LOO. It offers direct access to and visualization of the LDS, facilitating the identification and diagnosis of mechanical obstructions or pathologies [42]. After diagnosis, recanalization can be performed through direct or sheath-guided endoscopic probing [24, 25], or with the aid of a lacrimal trephine, especially for dense and fibrotic obstructions [31]. While DE-guided recanalization is most often performed using saline-irrigated DE, pressure-controlled air-insufflated HDDE can provide significantly better image quality, including both resolution and contrast, due to its ability to differentiate vascular and avascular areas. This is particularly for identifying obstructed sites, which are often avascular lesions surrounded by vascularized mucosa, and for clearing blood for better visibility [27]. In terms of the diagnostic outcomes, DE enabled the diagnosis of the types, causes, locations and patterns of LOO in 33.3% [24–26], 44.4% [26, 30, 32, 34, 37, 38, 40, 41] and 5.6% [34] of the included studies. The types or causes of LOO were generally classified into structural (stenosis, fibrosis) and secretory (mucus, stones, granulations) [24, 32, 34], or simple (membrane, cleft) and complicated (stenosis, fibrosis) [39]. DE could also identify the causes of probing failure [26] and detect signs of chronic inflammation (ectasia and hyperaemia) of LOO [25]. These findings allow for early diagnosis, prevention and management of probing failure, and timely treatment to prevent disease progression from simple obstructions to secondary inflammation (chronic dacryocystitis) or infection (acute dacryocystitis) [25]. The locations of LOO could be classified into pre-sac (canaliculus, sac) and post-sac (NLD, IM) [32, 34], or lower (membranous) and higher (sac-duct junction) [40]. The patterns of LOO were classified into focal and diffuse [34]. However, due to the varied definitions of types, causes and locations of LOO in different studies, and only one study classified LOO according to patterns, it was not possible to draw conclusions about the most common presentation of LOO.

As for therapeutic outcomes, DE-guided recanalization demonstrated success rates ranging from 73.7% [32] to 100% [26, 33, 39, 41], with 78.6% of studies reporting both objective improvements (anatomical and/or functional patency) and subjective benefits (symptom relief, improved Munk’s scores, interviews, or questionnaires). Success rates were generally higher in pediatric populations CNLDO (77.8% [25] to 100% [26, 33, 39, 41]) than in adults with PANDO or other LOO (73.7% [32] to 96.9% [28]). Among pediatric cases, refractory CNLDO achieved the highest success rates (97.1% [30] to 100% [26, 33]), while non-refractory complex CNLDO had the lowest (77.8% [25]). In adults, non-refractory PANDO achieved high success rates (84.9% [36] to 95.6% [31]), while the lowest adult success rates were observed in PANDO with false passages (73.7% [32]). These findings highlight the method’s high efficacy, especially in refractory cases, while also indicating challenges in managing complex obstructions such as non-refractory complex CNLDO and PANDO with false passages.

To enhance therapeutic success, 50% studies used adjuvants, such as topical steroids and antibiotics irrigated into the LDS at the end of procedure, to control inflammation and prevent infection [25, 26, 28, 31, 34–37, 41]. Systemic [34, 35] or nasal [25, 26] medications also played a role. Continuous saline irrigation of the LD to remove any remaining obstructions was crucial in maintaining LDS patency and preventing re-obstruction or recurrence, especially in patients with more severe disease, (i.e. higher degree of obstruction, longer symptom duration, or more resistance to previous treatment) [28, 36, 37]. The enhanced success rates were more evident in adult than pediatric populations. In adults with PANDO or other LOO, those who used adjuvants had higher success rates compared to those who did not (84.9% [36] to 95.6% [31] versus 73.7% [32] to 86.2 [24]), while the use of topical ophthalmic medications alone yielded the highest success rates [31]. In pediatric populations with CNLDO, 62.5% of studies (five out of eight) did not mention the use of adjuvants [29, 30, 33, 38, 39]. These studies had success rates ranging from 88.4% [38] to 100% [33, 39]. Two studies targeting patients with complex or refractory CNLDO used nasal medications, with success rates of 77.8% [25] and 100% [26], respectively. While one study who used topical antibiotics had 100% success rate [41].

DE-guided recanalization demonstrates not only high diagnostic accuracy and therapeutic success rates but also low complication rates, making it a highly effective and safe procedure. A significant percentage of studies (44.4%) reported no complications [25]; while only 16.7% noted minor issues, such as hematoma, edema, and surrounding soft tissue inflammation [35, 36, 38]. While 22.2% of studies reported the formation of false passages [26, 28, 30, 32], these were effectively managed [24, 32].

Compared to the traditional treatment methods like EXT-DCR or END-DCR, DE-guided recanalization offers several advantages, including shorter treatment times, faster recovery, lower complication rates, and better cosmetic outcomes [42]. Additionally, DE plays a crucial role in the diagnosis and treatment of complex or refractory CNLDO, canalicular stenosis, and PANDO by providing direct visualization of the LDS, precisely identifying obstructions, and enabling effective recanalization [43]. Our systematic review has several limitations. First, most of the included studies focused on Asian population (83.3% (15 out of 18)) and had a high female-to-male ratio (81.3% (13 out of 16)). Ethnic and sex variations in the anatomical structures of LDS [44] may influence the diagnostic and therapeutic success of DE-guided recanalization. Caution should be taken when applying these results to other populations. Second, the FU duration of the included studies ranged from 2 weeks [41] to 24.8 ± 15.5 months [25], which may not be long enough to establish long-term significance. Although the studies reported high therapeutic success and low complication rates, long-term outcomes and safety issues may be underreported or inconclusive. Future studies with longer FU periods are needed. Third, most studies (94.4% (17 out of 18)) were classified as Level III evidence and Level B in terms of importance of application. Future studies with Level I evidence would be needed to directly compare the efficacy and safety of DE-guided recanalization with traditional treatments of LOO, or to compare different types of DE and stents, and to evaluate various types, locations, patterns, and severities of LOO using more standardized definitions.

In addition to the limitations of our review, DE-guided recanalization itself is associated with high operative costs, a steep learning curve, and variable success rates [45]. Future research should focus on cost management, resource allocation, advanced training, and further supportive studies to optimize the management of patients with LOO.

## Conclusions

DE-guided recanalization has proven to be a highly effective procedure for both diagnosing and treating LOO, offering several advantages over traditional methods like EXT-DCR and END-DCR, including shorter treatment durations, quicker recovery times, and superior cosmetic outcomes. The procedure shows high success rates, especially in refractory cases. However, challenges persist in managing complex obstructions and avoiding false passages. The use of adjuncts such as topical steroids and antibiotics further improves therapeutic success, particularly in more severe cases. Future RCTs with standardized definitions, longer FU periods, and more diverse patient populations are needed to confirm and refine these findings.

## Supplementary Information

Below is the link to the electronic supplementary material.

Supplementary file1 (JPEG 179 KB)

Supplementary file2 (XLSX 28 KB)

## Data Availability

No datasets were generated or analysed during the current study.

## Abbreviations

LOO: Lacrimal outflow obstruction
DCR: Dacryocystorhinostomy
EXT-DCR: External dacryocystorhinostomy
END-DCR: Endonasal dacryocystorhinostomy
STI: Silicone tube intubation
DE: Dacryoendoscopy
LD: Lacrimal duct
ELDR: Endoluminal lacrimal duct recanalization
LDS: Lacrimal drainage system
CNLDO: Congenital nasolacrimal duct obstruction
PANDO: Primary acquired nasolacrimal duct obstruction
CO: Canalicular obstruction
FU: Follow-up
RCT: Randomized controlled trial
PPP: Preferred practice pattern
SIGN: Scottish intercollegiate guideline network
GRADE: Grading of recommendations assessment, development and evaluation
RCTs: Randomized controlled trials
LOO-NOS: Lacrimal outflow obstruction-not otherwise specified
3CCD: Three-charge-couple device
STs: Silicone tubes
NE: Nasoendoscope
IM: Inferior meatus
NLD: Nasolacrimal duct
CDE: Conventional dacryoendoscopy
HDDE: High-definition dacryoendoscopy
LIT: Lacrimal irrigation test
FDDT: Fluorescein dye disappearance test
FEDT: Functional endoscopic dye test
FDRT: Fluorescein dye retention test
TMH: Tear meniscus height
SE-DCR: Standard external-dacryocystorhinostomy
GBI: Glasgow benefit inventory
NEI VFQ-25: National Eye Institute 25-item visual function questionnaire
