# Supplementary figures and images for: Dacryoendoscopy in patients with lacrimal outflow obstruction: a systematic review
Source: Int Ophthalmol. 2025 Mar 14;45(1):90. doi: 10.1007/s10792-024-03388-z (PMC11909073; doi:10.1007/s10792-024-03388-z)

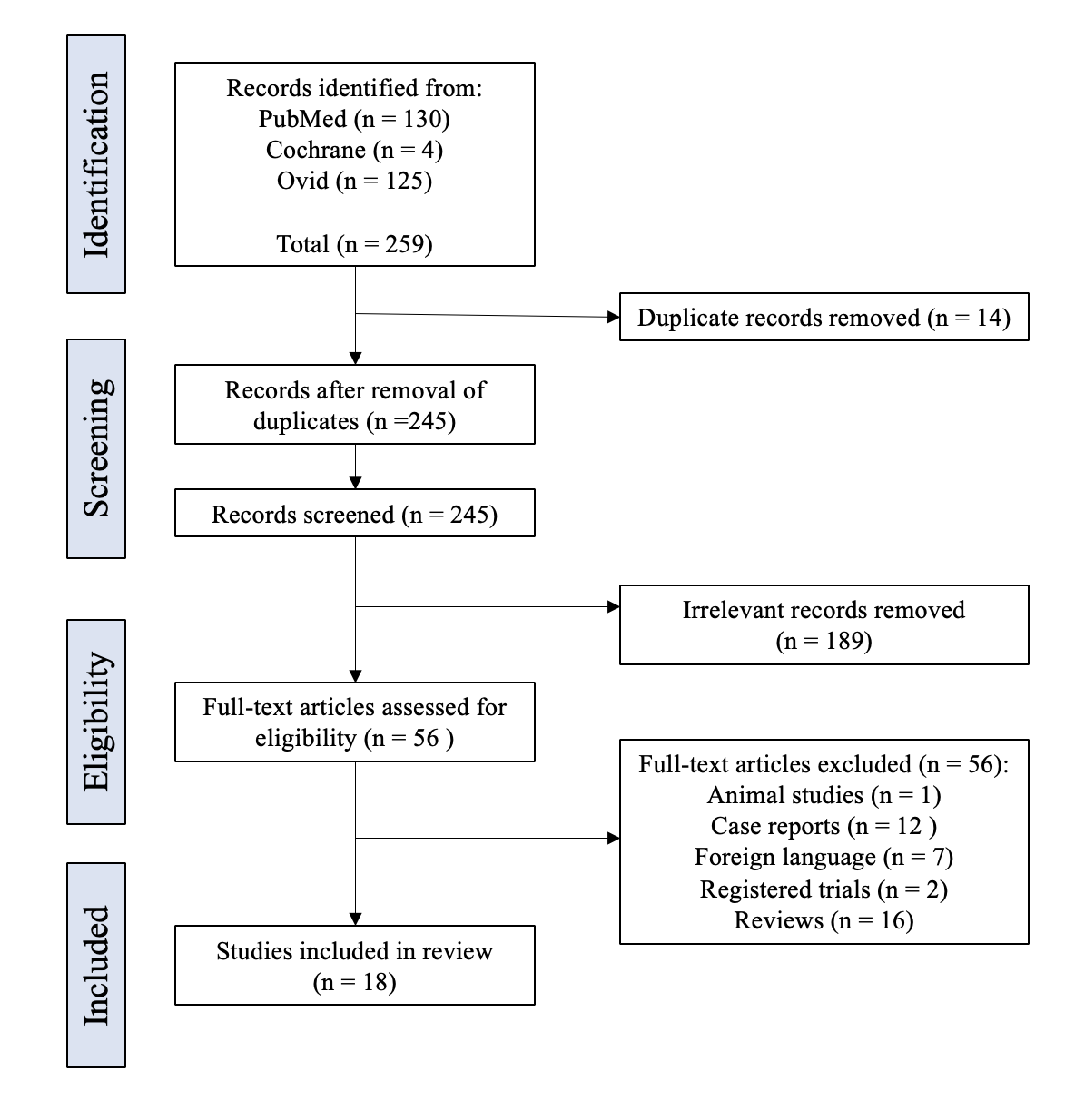

Supplement: Supplementary file 1 — Supplementary file1 (JPEG 179 KB) [file 10792_2024_3388_MOESM1_ESM.jpeg]
